# MicroRNA-485-5p suppresses the proliferation, migration and invasion of small cell lung cancer cells by targeting flotillin-2

**DOI:** 10.1080/21655979.2019.1586056

**Published:** 2019-03-05

**Authors:** Feng Gao, Hao Wu, Rui Wang, Yang Guo, Zefeng Zhang, Tao Wang, Guoliang Zhang, Changjiang Liu, Junfeng Liu

**Affiliations:** a Department of Thoracic Surgery, the Fourth Hospital of Hebei Medical University, Shijiazhuang, China; b Department of Clinical Laboratory, Hebei Medical University, Shijiazhuang, China

**Keywords:** miR-485-5p, FLOT2, small cell lung cancer, proliferation, metastasis

## Abstract

This study is aimed to elucidate the mechanisms underlying the role of miR-485-5p in small cell lung cancer (SCLC). The expression of miR-485-5p were quantified with real time quantitative PCR and it was found that the level of miR-485-5p was lower in SCLC tissues than normal tissues. In cultured SCLC cell lines, overexpression of miR-485-5p reduced cell proliferation, migration, and invasion in vitro, whereas knockdown of miR-485-5p performed contrary. FLOT2 expression was obviously upregulated and negatively correlated with miR-485-5p expression level in SCLC tissues. Overexpression of miR-485-5p significantly inhibited the protein expression of flotillin-2 (FLOT2) in cultured SCLC cells. Luciferase reporter assay confirmed that FLOT2 was a direct target of miR-485-5p in SCLC cells. It is concluded that miR-485-5p, as a tumor suppressor, inhibits the growth and metastasis in SCLC by targeting FLOT2. Upregulation of miR-485-5p expression may be an attractive strategy for SCLC therapy.

## Introduction

Lung cancer is one of the malignant tumors that have the fastest-growing incidence rate, serious morbidity and high rate of mortality, threatening the health and life of people all over the world [1–3]. Small cell lung cancer (SCLC) is one of the major types of lung cancer, accounting for approximately 20%-25% of lung cancer. SCLC has the pathological characteristics of high degree of malignancy, low degree of differentiation, rapid growth, invasion of blood vessels, early and extensive metastasis with poor biological behavior and dangerous prognosis. Moreover, SCLC patients are less symptomatic before diagnosis and survive shorter than patients with other types of lung cancer [4–6]. Although a number of studies have demonstrated that many molecular triggers play a vital role in the development of SCLC, the mechanisms underlying the process remain unclear. An understanding of these mechanisms is crucial for developing effective treatments for this disease.

MicroRNAs (miRNAs) are endogenous small-noncoding RNAs that can silence their cognate target genes usually by imperfect base-pairing to the 3′ untranslated region (UTR) of a target mRNA, which results in either mRNA degradation or translation inhibition [7,8]. miRNAs play important roles in the regulation of various cellular processes, including cell proliferation, differentiation, apoptosis and mobility [9–15]. The deregulation of miRNAs has been reported in many cancers, including lung, gastric [16], colorectal [17–20], and liver cancers [21], as well as leukemia [22] and lymphoma [23,24]. Furthermore, in a context-dependent manner, miRNAs can function either as oncogenes or tumor suppressors in tumor progression [10,11]. Therefore, miRNA expression profiles can be used as molecular biomarkers for cancer diagnosis, classification, clinical prognostic information and therapy [11–14].

Flotillin (Flot) is a protein family on microdomain lipid rafts, which have been reported to play a role in various biological processes, including cell survival, proliferation, adhesion, apoptosis, and motility, mainly due to its implication in vesicular invaginations of the plasma membrane, signal transduction pathways, organization of the cytoskeleton, protein sorting during both exocytosis and endocytosis, as well as synaptic transmission [15–20]. The Flotillin family contains two homologous isoforms, flotillin1 (FLOT1) and 2 (FLOT2), which play essential physical roles in various cellular processes such as adhesion, reorganization of the actin cytoskeleton, endocytosis, phagocytosis, and transduction of cellular signals. Flotillin-1 oligomerizes to build microdomain scaffolds that are involved in molecular sorting [21–23], cytoskeletal dynamics [24], clathrin-independent endocytic pathways [25–27] and phagosome trafficking [14–18]. However, it also promotes cell proliferation [22] and T cell activation [22,23]. Moreover, flotillin-1 functions as a regulatory signaling molecule that coordinates a variety of signal transduction processes [24]. Together with FLOT1, FLOT2 is a marker for caveolae lipid raft domains that tether growth factor receptors linked to signal transduction pathways. FLOT2 is important for non-caveolar raft formation and associated with the development and progression of cancer. Previous studies demonstrated that microRNAs can regulate the expression level of flotillin [25]. Butz H et al proved that validated CAV1 and FLOT1 as miR-124-3p targets [26]. Huang et al showed that FLOT2 identified as a direct target of miR-133 in human lung adenocarcinoma [27]. And Wang et al identified Flot2 as a direct target of miR-802 in PCa cells [28]. It is known that FLOT2 is upregulated in several types of cancer, including SCLC. It has been reported that miR-485-5p is an important regulator in many human cancers. miR-485-5p can target specific genes, such as IGF2BP2, and regulate proliferation, migration and metastasis in SCLC [29–31].However, the actual relationship between miR-485-5p and FLOT2 in SCLC needs to be well elucidated. In this study, we sought to determine whether miR-485-5p plays a functional role in the development and progression of SCLC by regulating FLOT2. Our results demonstrated that miR-485-5p acted as a tumor suppressor by directly targeting FLOT2, not FLOT1.

## Materials and methods

### Subjects and tissue specimens

This study was approved by the Ethics Committee of the Fourth Hospital of Hebei Medical University (Shijiazhuang, Hebei, China). All procedures on the participants including 18 SCLC patients and 56 healthy people were performed after a written consent was obtained from all of the subjects or guardians. In this study, SCLC tissues and adjacent tissues surgically resected from 18 SCLC patients. Normal lung biopsies were surgically collected from 56 healthy people. The samples were fixed in RNA later (Ambion, Austin, TX, USA) immediately after surgical resection and stored at −80°C in a freezer until subsequent use. All specimens were histologically and clinically diagnosed at the Department of Thoracic Surgery, the Fourth Hospital of Hebei Medical University.

### Cell culture

The human SCLC cell lines NCI-H446 and NCI-H1688 were purchased from the American Type Culture Collection (ATCC, Rockville, MD, USA). The cells were cultured in RPMI 1640 (Invitrogen, Beijing, China) supplemented with 10% fetal bovine serum (FBS, Invitrogen, Carlsbad, CA, USA). All cells were incubated at 37°C in a humidified incubator (Thermo Electron Corp, New Castle, DE) containing 5% CO_2_.

### Transfection

The NCI-H446 and NCI-H1688 cell lines were transfected with 100 nM miR-485-5p inhibitor, miR-485-5p mimics, miRNA mimic negative controls (NC miR-mimics) and miRNA inhibitor negative controls (GenePharma, Shanghai, China) using Lipofectamine® 2000 (Invitrogen; Thermo Fisher Scientific, Waltham, MA, USA), according to the manufacturer’s instructions.

### RNA extraction and quantitative real-time PCR

RNA extraction and quantitative real-time PCR (RT-qPCR) were carried out according to the protocols previously reported [32]. Total RNA was extracted from tissues and cultured cells using TRIzol Reagent (Invitrogen, CA, USA). cDNA was synthesized from 2 μg of total RNA with random primers using Omniscript reverse transcription kit (Qiagen). RT-qPCR was subsequently performed in triplicate to measure miR-485-5p or FLOT2 mRNA with a 1:4 dilution of cDNA using the QuantiTect SYBR green PCR system (Qiagen). Gene-specific q-PCR primers were as follows: U6 snRNA reverse transcript primer: 5ʹ-CGCTTCACGAATTTGCGTGTCAT-3ʹ, U6 snRNA forward primer: 5ʹ-GCTTCGGCAGCACATATACTAAAAT-3ʹ;U6 snRNA reverse primer: 5ʹ-CAGTGCGTGTCGTGGAGT-3ʹ. miR-485-5p reverse transcript primer: 5ʹ-GTCGTATCCAGTGCGTGTCGTG GAGTCGG CAATTGC ACTGGATACGACGAATTCA-3ʹ, miR-485-5p forward primer: 5ʹ-GGAGAGGCTGGCCGTGAT-3ʹ; miR-485-5p reverse primer: 5ʹ-CAGTGCGTGTCGTGGAGT-3ʹ. All primers were purchased from Qiagen. The relative expression levels of miRNA and mRNA were normalized to the expression of U6 snRNA. Data were collected and analyzed with the Rotor-Gene software accompanying the PCR machine. The relative mRNA expression levels were expressed as 2^−△△Ct^.

### Cell proliferation assay

Twenty-four hours after transfection with miR-485-5p inhibitor, miR-485-5p mimics, miRNA mimic negative controls (NC miR-mimics) and miRNA inhibitor negative controls, NCI-H446 and NCI-H1688 cells were harvested and sub-cultured in 96-well plates. Cell proliferation was assessed using thiazolyl blue tetrazolium bromide assay (MTT; Amresco, Radnor, PA, USA) according to the manufacturer’s instructions. Briefly, MTS reagent (20 µl) was added to each well and incubated at 37°C for 4 h. Then, the reagent was removed and dimethyl sulfoxide (150 µl) was added to each well. Absorbance at 492 nm was measured using the FLx800 fluorescence microplate reader (BioTek, Winooski, VT, USA). The experiment was performed in triplicate and repeated three times. The data are expressed as means ± standard error of the mean (SEM).

### Trypan blue exclusion test of cell viability

Cells were seeded at a density of 2 × 10^4^ cells/well in 24-well plates and incubated with miR-485-5p inhibitor, miR-485-5p mimics, miRNA mimic negative controls (NC miR-mimics) and miRNA inhibitor negative controls. Cells were harvested after 24, 48, 72 and 96 h of incubation. Trypan blue solution (Gibco, USA) was added to the cell suspensions in a ratio of 1:1. The numbers of total cells and dead cells (stained in blue) were counted using hemocytometer. No less than 100 cells were counted each time for three times to achieve an average. The percentage of living cells and dead cells was calculated based on the formula: total cells = living cells + dead cells. The experiments were performed in triplicate, and data were expressed as means ± SE.

### Western blotting

Proteins were extracted from the SCLC tissues, paired non-tumor lung tissues and cultured cells with RIPA lysis buffer (1% NP40, 0.1% sodium dodecyl sulfate (SDS), 100 μg/ml phenylmethylsulfonyl fluoride, and 0.5% sodium deoxycholate, in PBS) on ice. The supernatants were collected after centrifugation at 12000 × g at 4°C for 20 min. After the protein concentration was determined using a BCA protein assay kit (Bio-Rad, China), the lysates were mixed with 4 × SDS loading buffer (125 mmol/l Tris-HCl, 4% SDS, 20% glycerol, 100 mmol/l DTT, and 0.2% bromophenol blue) at a ratio of 1:3. After the samples were heated at 100 C for 5 min proteins were differentiated on SDS-polyacrylamide gels. The separated proteins were then transferred to a PVDF membrane. The membrane blots were probed with a primary antibody of rabbit IgG anti-FLOT1 (1:1,000), rabbit IgG anti-FLOT2 (1:1,000) or rabbit IgG anti-glyceraldehyde 3-phosphate dehydrogenase (1:1,000) (Cell Signaling Technology Inc., Boston, MA, USA) and horseradish peroxidase-conjugated anti-rabbit IgG (Jackson ImmunoResearch Labs, West Grove, PA, USA) secondary antibody, and developed with the enhanced chemiluminescent system. The signals were recorded using X-ray film. GAPDH was used as an internal reference for protein FLOT1 and FLOT2.

### Dual luciferase reporter assay

Luciferase reporters were successfully constructed using molecular cloning technology. FLOT2 mRNA 3′-UTR target sequence clone was purchased from Creative Biogene (Shirley, NY, USA). miR-485-5p-transfected NCI-H446 cells were seeded in 24 well plates for 24 h, then the cells were transfected with 1 μg of luciferase reporter plasmids per well. Luciferase activities were measured using the dual luciferase reporter gene assay kit (Promega, Beijing, China), according to the manufacturer’s instructions.

### Cell migration and invasion assays

The cell migration assay was performed using Transwell inserts (Corning, Blacksburg, VA, USA), and the cell invasion assay was performed with the CytoSelect 24-well cell invasion assay kit (Cell Biolabs, Inc., San Diego, CA, USA), according to the manufacturer’s instructions. The cells were transiently transfected as previously described [11] to overexpress or knock down miR-485-5p. At 24 h after transfection, cells were starved in RPMI 1640 without FBS for 2 h, and 6 × 10^4^ cells were suspended in 0.2 ml RPMI 1640 without FBS and seeded in the upper chamber of a Transwell insert. Then, RPMI 1640 containing 10% FBS was added to the lower chamber as a chemoattractant. When the cells in the upper chamber were cultured for 8 h at 37°C in humidified air containing 5% CO_2_ the cells that had migrated to the lower chamber were fixed and stained with 0.1% crystal violet. Three low-magnification fields (x200) were randomly selected, and the number of migrated or invasive cells was counted. All of the experiments were performed in triplicate, and data were expressed as means ± SE.

### Immunostaining

Immunostaining was performed according to literature [33]. Sections of SCLC tissues were made, detected with primary antibody against FLOT2 and enzyme-labelled secondary antibody, developed, mounted, and observed with microscopy. The total number of tumor cells and the number of positive cells were counted from five randomly selected high magnification fields (×400) under microscope. The percentages of positive cells were calculated, and designated ≤25% as 0 points, 26−50% as 1 point, 51–75% as 2 points, and > 75% was 3 points. The staining intensity was scored with no color development as 0 points, light tan color as 1 point, brownish yellow as 2 points, and tan color as 3 points. The sum of the two scores for each sample was calculated, with 0 points referred as (-), 1–2 points as (+), 3–4 points as (++), and 5–6 points as (+++). Samples with (++) and (+++) were defined as positive expression; samples with (+) or (-) were defined as negative expression. All the tests were independently performed and interpreted by two pathologists.

### Statistical analysis

SPSS software was used for statistical analysis. For comparison among the paired samples, paired t-test was performed. For comparison among the unpaired samples t-test was performed. The correlation between miR-485-5p and FLOT2 expression was analyzed using Spearman correlation analysis. All data were expressed as mean ± SD from at least three separate experiments.

## Results

### miR-485-5p expression in SCLC tissues

**Figure 1.**
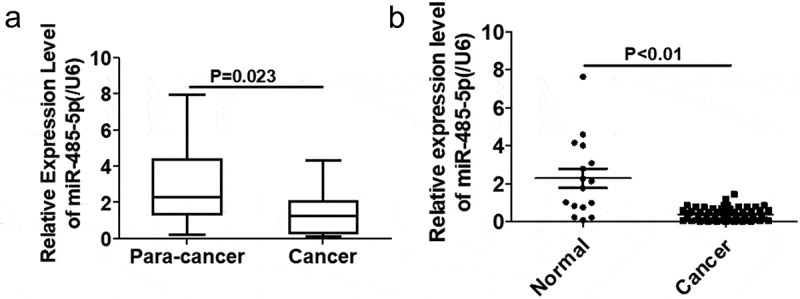
MiR-485–5p expression in SCLC tissues. (a). Comparison of the expression levels of miR-485-5p between SCLC tissues and the adjacent normal tissues. The expression levels of miR-485-5p in the SCLC tissues and the corresponding adjacent normal tissues from SCLC patients were detected by RT-qPCR using β-actin mRNA as an internal standard (n = 18). (b). Comparison of the expression levels of miR-485-5p between SCLC tissues from patients (n = 18) and normal tissues from healthy people (n = 56). miR-485-5p levels were detected by RT-qPCR using U6 RNA as an internal standard (p<0.01). Values were expressed as mean ± SD.

To investigate if expression of miR-485-5p was altered in SCLC, miR-485-5p expression levels in SCLC tissues and normal tissues were quantified using qPCR. The expression of miR-485-5p was significantly (p < 0.05) reduced in cancer tissues compared to adjacent normal tissues (Figure 1(a)). miR-485-5p expression was significantly decreased in SCLC tissues from SCLC patients (p < 0.01) as compared to the normal tissues from healthy people (Figure 1(b)).

### Effects of miR-485-5p on SCLC cell proliferation, migration and invasion

**Figure 2.**
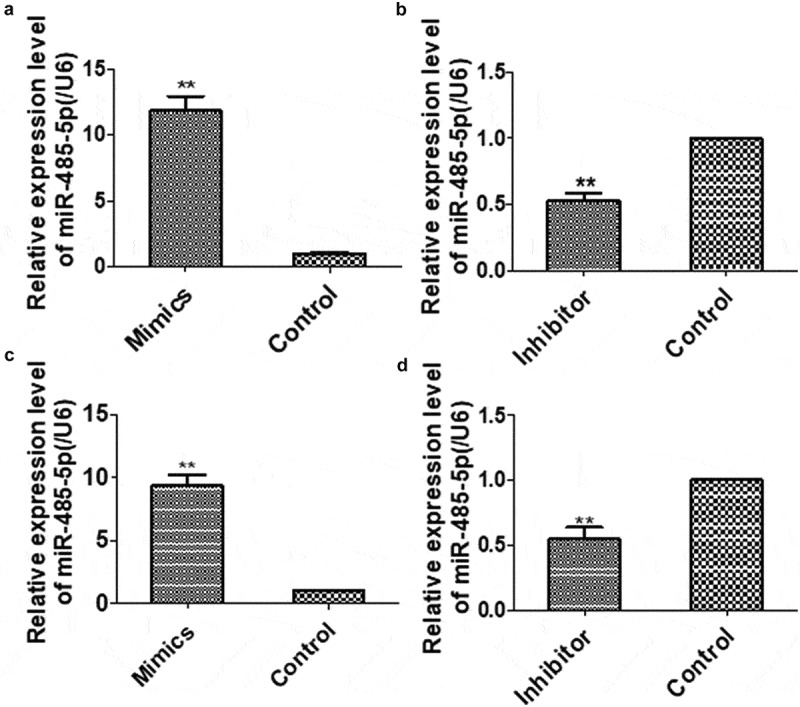
The levels of MiR-485-5 level in NCI-H446 and NCI-H1688 cells transfected with miR-485-5p mimics, miR-485-5p inhibitor or their corresponding controls. The expression levels of miR-485-5p were normalized to U6 RNA expression and were presented as the mean ± SEM from three independent experiments. (a), NCI-H446 cells were transfected with miR-485-5p mimic or mimimics-NC; (b), NCI-H446 cells were transfected with miR-485-5p inhibitor or the corresponding control; (c), NCI-H1688 cells were transfected with miR-485-5p mimics or mimics-NC; (d), NCI-H1688 cells were transfected with miR-485-5p inhibitor or the corresponding inhibitor control (n=3, **p<0.01).

To determine the effects of miR-485-5p on SCLC, the expression of miR-485-5p in SCLC cells were intervened.NCI-H446 and NCI-H1688 cells were transfected with miR-485-5p mimics, miR-485-5p inhibitor, miRNA mimic negative control or miRNA inhibitor negative control. qPCR showed that miR-485-5p level was significantly (p < 0.05) increased in miR-485-5p-transfected NCI-H446 (Figure 2(a)) and NCI-H1688 cells (Figure 2(c)), while transfection of the cells with miR-485-5p inhibitor significantly decreased (p < 0.05) miR-485-5p level by 50% of the control (Figure 2(b,d)). These results demonstrated that the miR-485-5p level was successfully altered in the SCLC cells by the transfection strategy.

**Figure 3.**
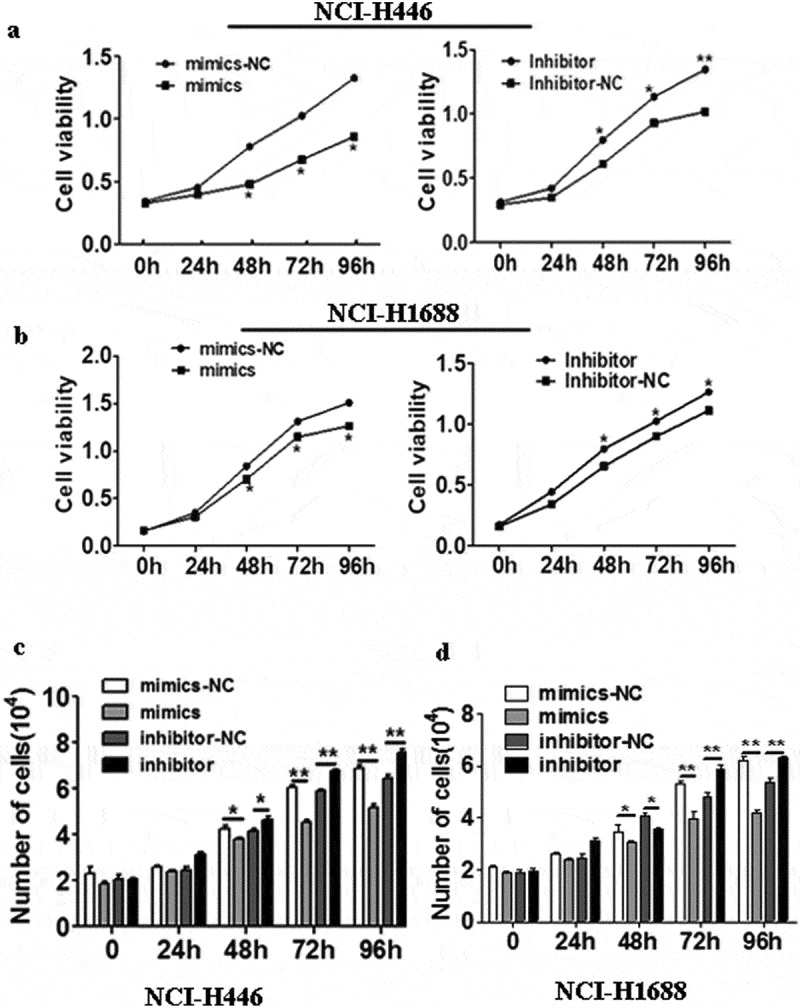
Effects of miR-485-5p and its inhibitor on the cell proliferation ability in NCI-H446 and NCI-H1688 cells. Cell viability was determined by MTS assay(a,b) and trypan blue exclusion assay(c,d) and expressed as mean ± SEM from three independent experiments. (a), NCI-H446 cells were transfected with miR-485-5p mimics or mimics-NC and miR-485-5p inhibitor or inhibitor-NC; (b), NCI-H1688 cells were transfected with miR-485-5p mimics or mimics-NC and miR-485-5p inhibitor or inhibitor-NC; (n = 3, *p < 0.05). (c), NCI-H446 cells were transfected with miR-485-5p mimics or mimics-NC and miR-485-5p inhibitor or inhibitor-NC; (d), NCI-H1688 cells were transfected with miR-485-5p mimics or mimics-NC and miR-485-5p inhibitor or inhibitor-NC; (n=3, *p<0.05).

The effects of the changes in miR-485-5p expression on SCLC cell lines NCI-H466 and NCI- H1688 were measured by MTS assay and trypan blue exclusion test. After the cells were transfected for 48h overexpression of miR-485-5p significantly (p < 0.05) decreased cell proliferation in NCI-H446 (Figure 3(a)) and NCI-H1688 cells (Figure 3(b)), whereas knockdown of miR-485-5p by its inhibitor significantly (p < 0.05) increased cell proliferation in NCI-H446 (Figure 3(a)) and NCI-H1688 cells (Figure 3(b)). We examined whether overexpression or knockdown of miR-485-5p increases cell death by using trypan blue exclusion test. We found that cells treated with miR-485-5p mimics significantly (p < 0.05) decreased the number of cell after indicated times of incubation in NCI-H446 (Figure 3(c)) and NCI-H1688 cells (Figure 3(d)), whereas cells treated with miR-485-5p inhibitor significantly (p < 0.05) increased the number of cells after indicated times of incubation in NCI-H446 (Figure 3(c)) and NCI-H1688 cells (Figure 3(d)). These results demonstrated that overexpression of miR-485-5p decreased cell proliferation by MTS assay and trypan blue exclusion test.

**Figure 4.**
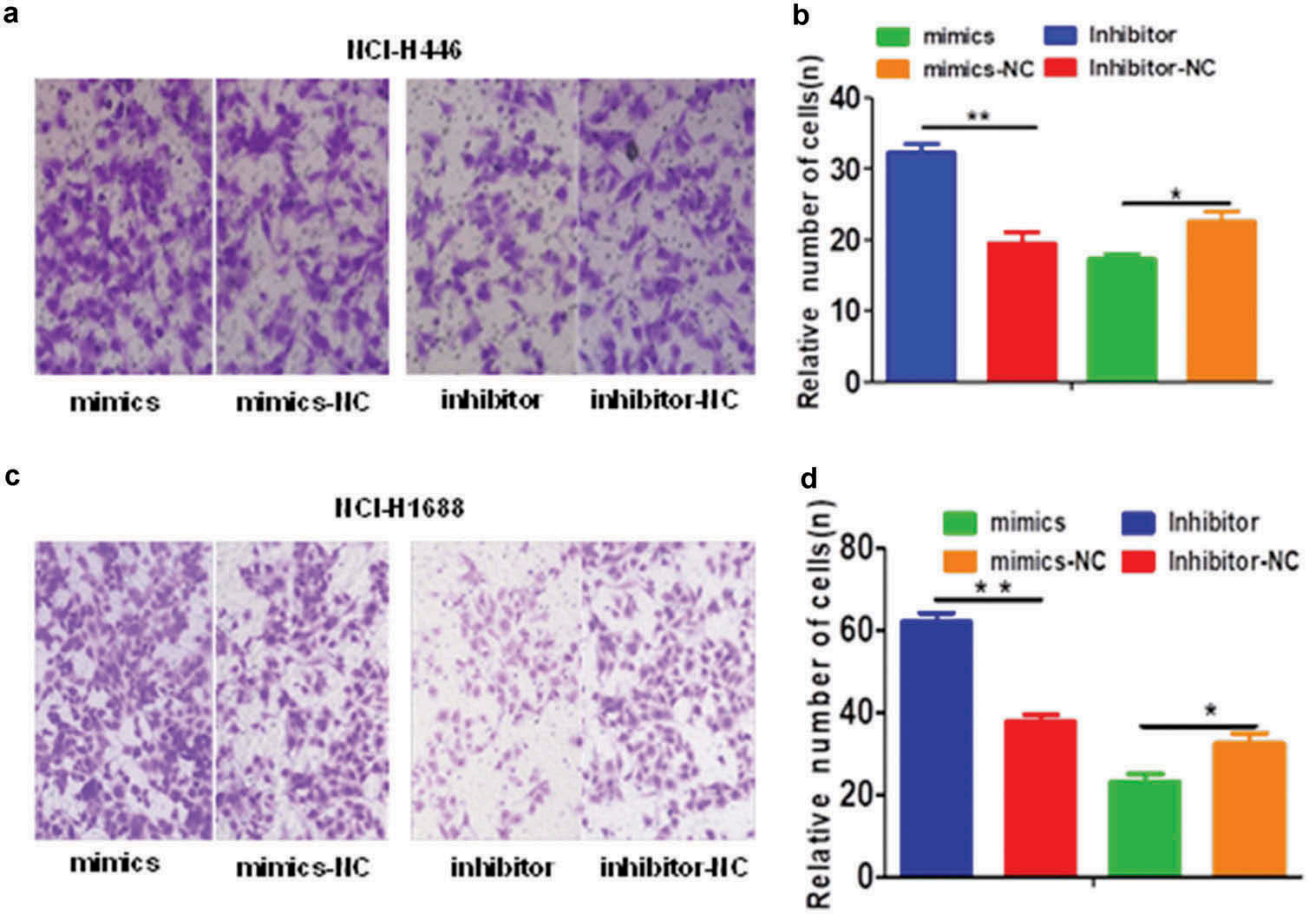
Effects of miR-485-5p and its inhibitor on the cell migration ability in NCI-H446 and NCI-H1688 cells. Cell migration was determined by Boyden chamber assay. The number of cells migrated across the membrane were quantified and expressed as mean ± SEM from three independent experiments. (a), Boyden chamber assay of NCI-H446 cells; NCI-H446 cells were transfected with miR-485-5p mimics, miR-485-5p inhibitor or their corresponding controls (mimics-NC, inhibitor-NC), respectively; (b), comparison of the number of migrated cells among the NCI-H446 cells transfected with miR-485-5p mimics, miR-485-5p inhibitor or their corresponding controls; (c), NCI-H1688 cells were transfected with miR-485-5p mimics, miR-485-5p inhibitor or their corresponding controls (mimics-NC, inhibitor-NC), respectively; (d), comparison of the number of migrated cells among the NCI-H1688 cells transfected with miR-485-5p mimics, miR-485-5p inhibitor or their corresponding controls (n=3, *p<0.05, **p<0.01).

**Figure 5.**
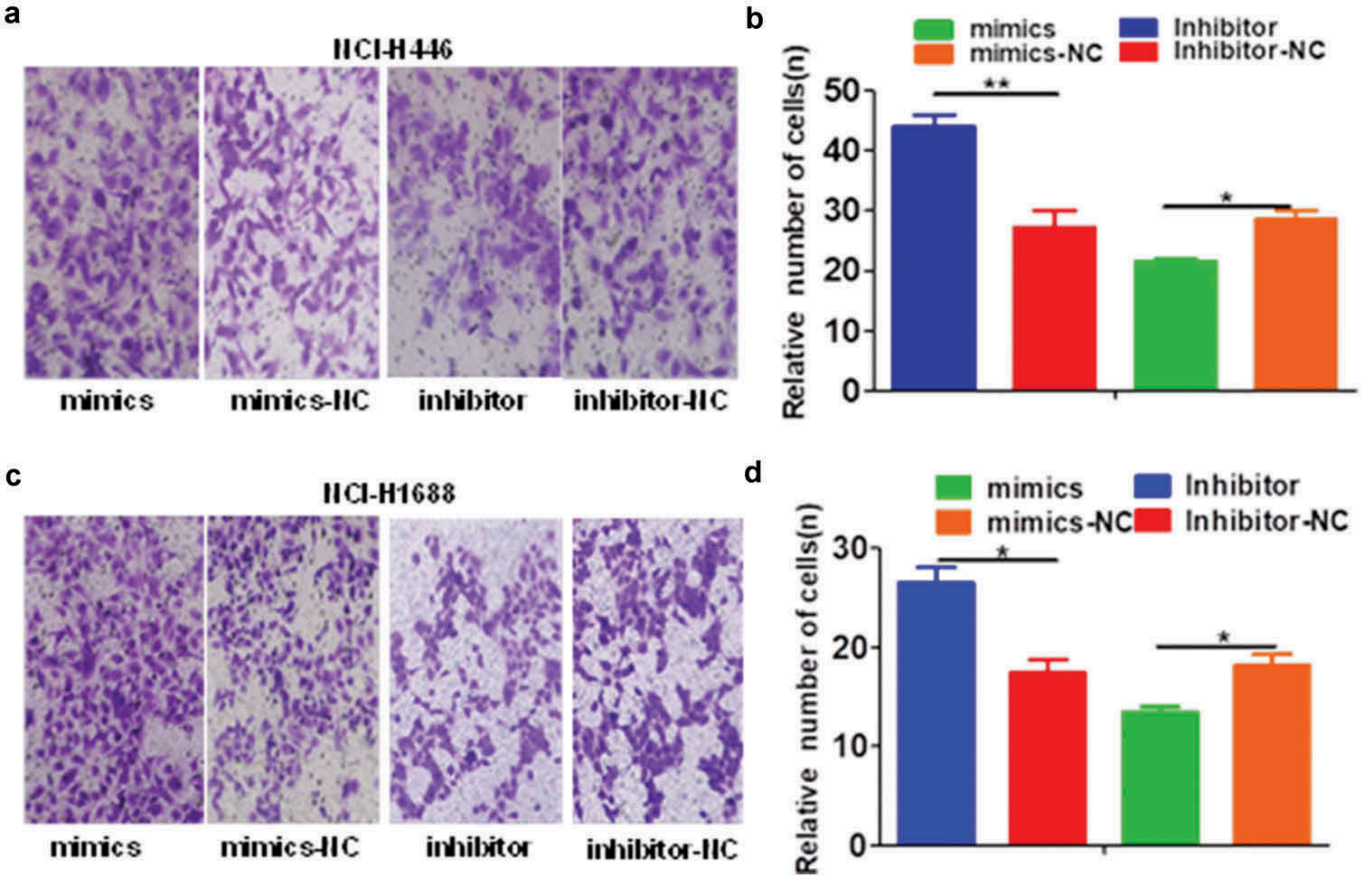
Effects of miR-485-5p and its inhibitor on the cell invasion ability in NCI-H446 and NCI-H1688 cells. Cell invasion ability of NCI-H446 and NCI-H1688 cells was determined by Transwell matrigel invasion assay. The number of cells invaded into the matrigel were quantified and expressed as mean ± SEM from three independent experiments. (a), Transwell matrigel invasion assay of NCI-H446 cells; NCI-H446 cells were transfected with miR-485-5p mimics, miR-485-5p inhibitor or their corresponding controls (mimics-NC, inhibitor-NC), respectively; (b), comparison of the number of invaded cells among the NCI-H446 cells transfected with miR-485-5p mimics, miR-485-5p inhibitor or their corresponding controls; (c), Transwell matrigel invasion assay of NCI-H1688 cells; NCI-H1688 cells were transfected with miR-485-5p mimics, miR-485-5p inhibitor or their corresponding controls (mimics-NC, inhibitor-NC), respectively; (d), comparison of the number of invaded cells among the NCI-H1688 cells transfected with miR-485-5p mimics, miR-485-5p inhibitor or their corresponding controls (n=3, *p<0.05, **p<0.01).

The effects of the changes in miR-485-5p expression on SCLC cell migration and invasion were further analyzed by cell migration and invasion assays. The number of NCI-H446 and NCI-H1688 cells that migrated across the membrane significantly (p < 0.05) increased in miR-485-5p inhibitor-transfected cells compared with those transfected with miRNA inhibitor negative control (Inhibitor-NC) or miR-485-5p mimics (Figure 4(a–d)). The number of NCI-H446 and NCI-H1688 cells that invaded into the matrigel significantly (p < 0.05) decreased in miR-485-5p mimic-transfected cells compared with those transfected with miRNA mimic negative control (mimics – NC) (Figure 5(a–d)). The number of NCI-H446 and NCI-H1688 cells that invaded into the matrigel significantly (p < 0.05) increased in miR-485-5p inhibitor-transfected cells compared with those transfected with miRNA inhibitor negative control (Inhibitor-NC) or miR-485-5p mimics (Figure 5(a–d)).

### Relationship between miR-485-5p and FLOT2

**Figure 6.**
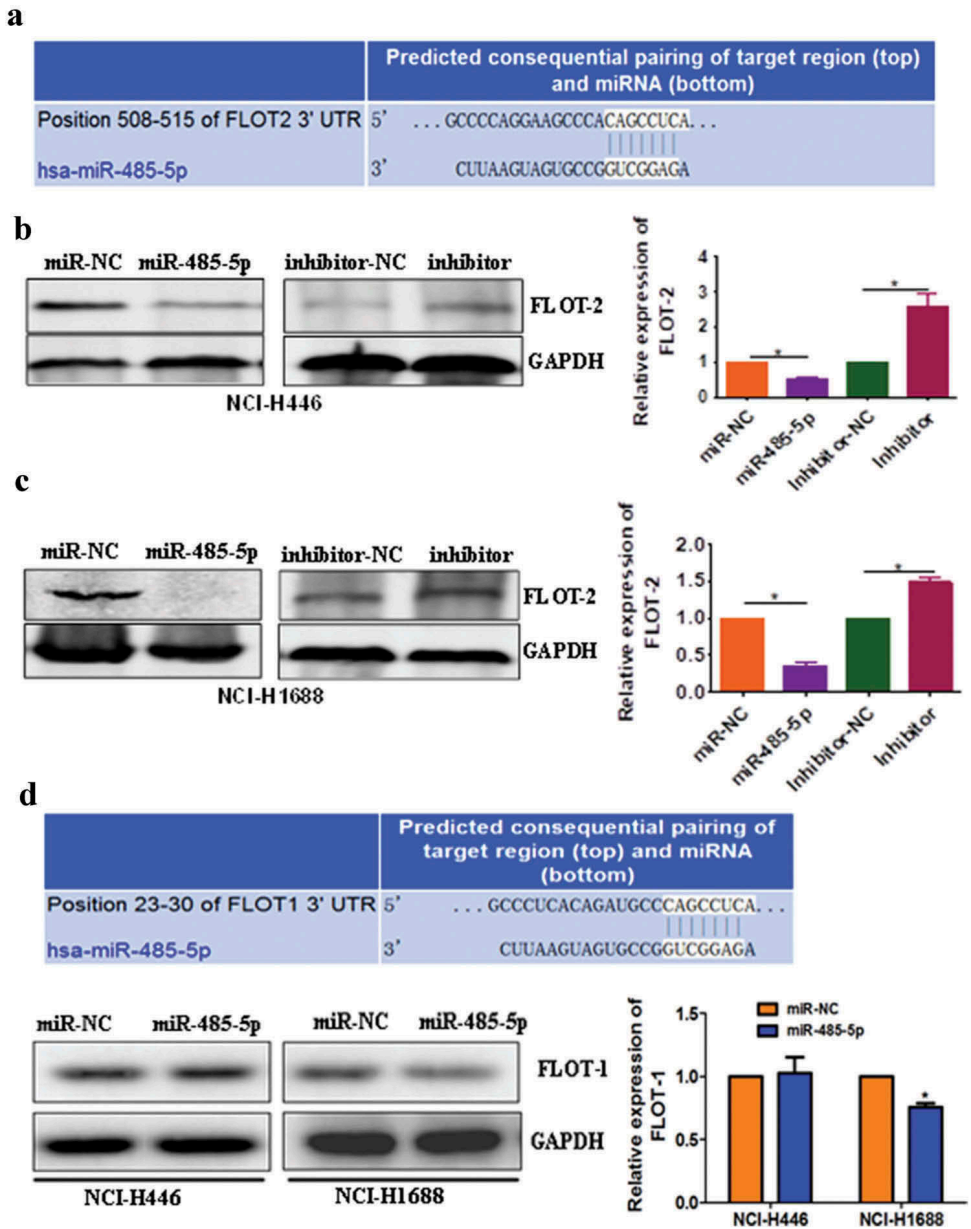
MiR-485-5p targets 3′-UTR of FLOT2 mRNA to inhibit its translation. (a), bioinformatics analysis revealed a miR-485-5p-binding site in the 3ʹUTR of FLOT2 mRNA. (b), Western blotting of FLOT2 protein in NCI-H446 cells transfected with miR-485-5p mimics, miR-485-5p inhibitor or their corresponding controls (miR-NC, inhibitor-NC), respectively; (c), comparison of FLOT2 protein levels among NCI-H1688 cells transfected with miR-485-5p mimics, miR-485-5p inhibitor or their corresponding controls; (d), bioinformatics analysis revealed a miR-485-5p-binding site in the 3ʹUTR of FLOT1 mRNA and Western blotting of FLOT1 protein in NCI-H446 cells and NCI-H1688 transfected with miR-485-5p mimics and miR-NC. FLOT2 protein levels were determined by Western blot assay and normalized to GAPDH. The data represent the mean ± SEM from three independent experiments (n = 3, *p < 0.05).

To determine if miR-485-5p targets FLOT2, bioinformatics analysis was performed to search for the putative mir-485-5p targeting sites in FLOT2 transcripts. It was found that FLOT2 3′-UTR contained a miR-485-5p-binding site, suggesting that FLOT2 could be a direct target of miR-485-5p (Figure 6(a)). To test if miR-485-5p influences the translation of FLOT2 mRNA, the effects of intervened miR-485-5p on FLOT2 protein level were analyzed. Western blot analysis showed that FLOT2 expression level was significantly (p < 0.05) decreased in miR-485-5p mimic-transfected NCI-H446 and NCI-H1688 cells compared with those transfected with mimic negative control (miR-NC) while silencing of miR-485-5p expression by its inhibitor (antagomir) markedly (p < 0.05) increased the expression level of FLOT2 protein compared with cells transfected with miRNA inhibitor negative control (inhibitor-NC) (Figure 6(b,c)). Furthermore, we also found that FLOT1 3′-UTR also contained a miR-485-5p-binding site (Figure 6(d)), suggesting that FLOT1 may also be a direct target of miR-485-5p. However, Western blot analysis showed that FLOT1 expression level was not decreased in miR-485-5p mimic-transfected NCI-H446 cells, though it was mildly decreased in NCI-H1688 cells (p < 0.05). These results indicated that miR-485-5p influenced FLOT2 protein level in both NCI-H446 cells and NCI-H1688 cells, while influenced FLOT1 protein level only in NCI-H1688 cells but not in NCI-H446 cells.

**Figure 7.**
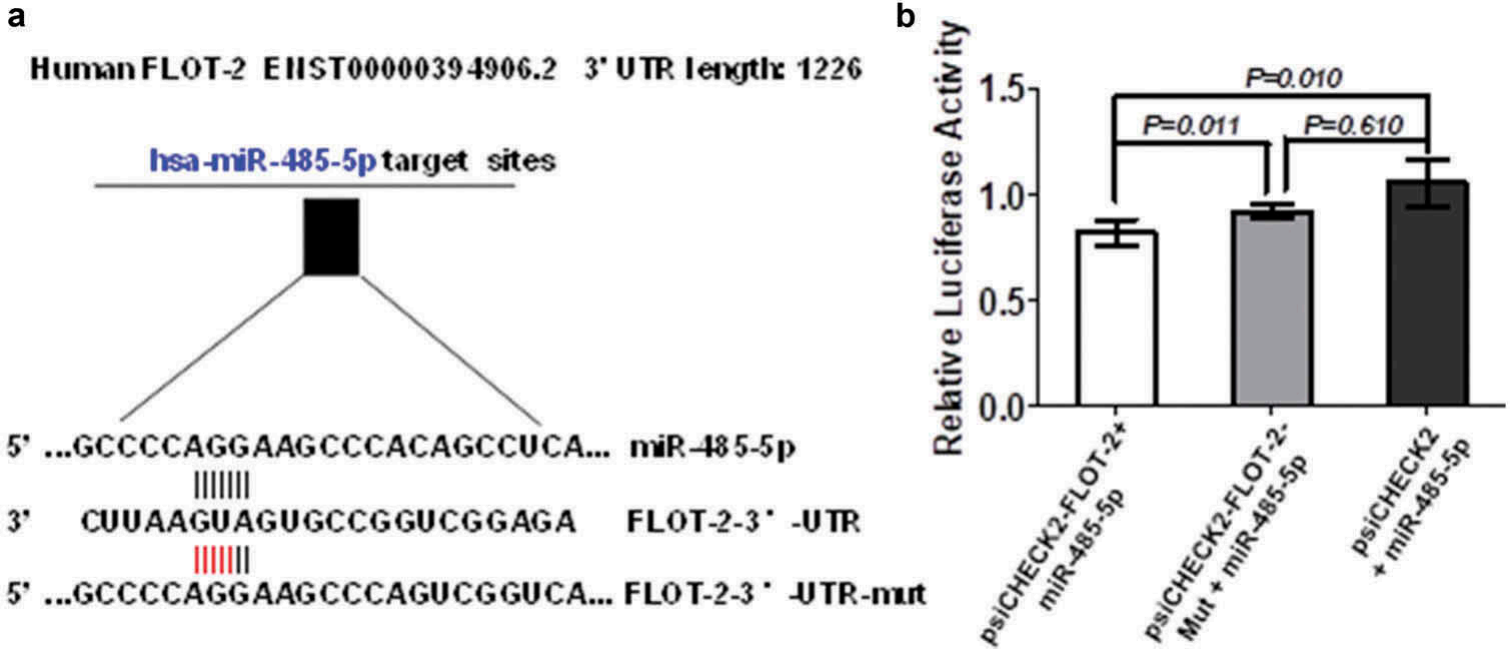
FLOT2 was directly targeted by miR-485-5p. (a), sequence of the miR-485-5p-binding site in the 3ʹUTR of FLOT2 mRNA and the sequence of the mutation introduced into the site; (b), effects of miR-485-5p on the translation of the reporter gene inserted downstream of the 3ʹUTR of FLOT2 mRNA or the mutated 3ʹUTR of FLOT2 mRNA in NCI-H446 cells. NCI-H446 cells were co-transfected with the indicated constructs and miR-485-5p, and luciferase activities were measured using the Dual Luciferase Reporter Kit. The luciferase activity was normalized and expressed as the ratio of firefly/Renilla luciferase activities. Data represent the mean ± SEM of triplicate experiments (n = 3, p < 0.05).

To further test if miR-485-5p regulated FLOT2 expression at post-transcriptional level by targeting its 3ʹ-UTR, we constructed the wild-type psiCHECK2-FLOT2-3′-UTR and its mutant psiCHECK2-FLOT2-3′-UTR-mut. Co-transfection of NCI-H446 cells with the wild-type psiCHECK2-FLOT2-3′-UTR and miR-485-5p mimics significantly decreased luciferase activity (p < 0.05), whereas co-transfection of the cells with mutant psiCHECK2-FLOT2-3′-UTR-mut and miR-485-5p mimics abolished the inhibitory effect of miR-485-5p on luciferase activity in the SCLC cells (Figure 7(a,b). These results confirmed that miR-485-5p inhibited FLOT2 translation by targeting its 3ʹ-UTR.

**Figure 8.**
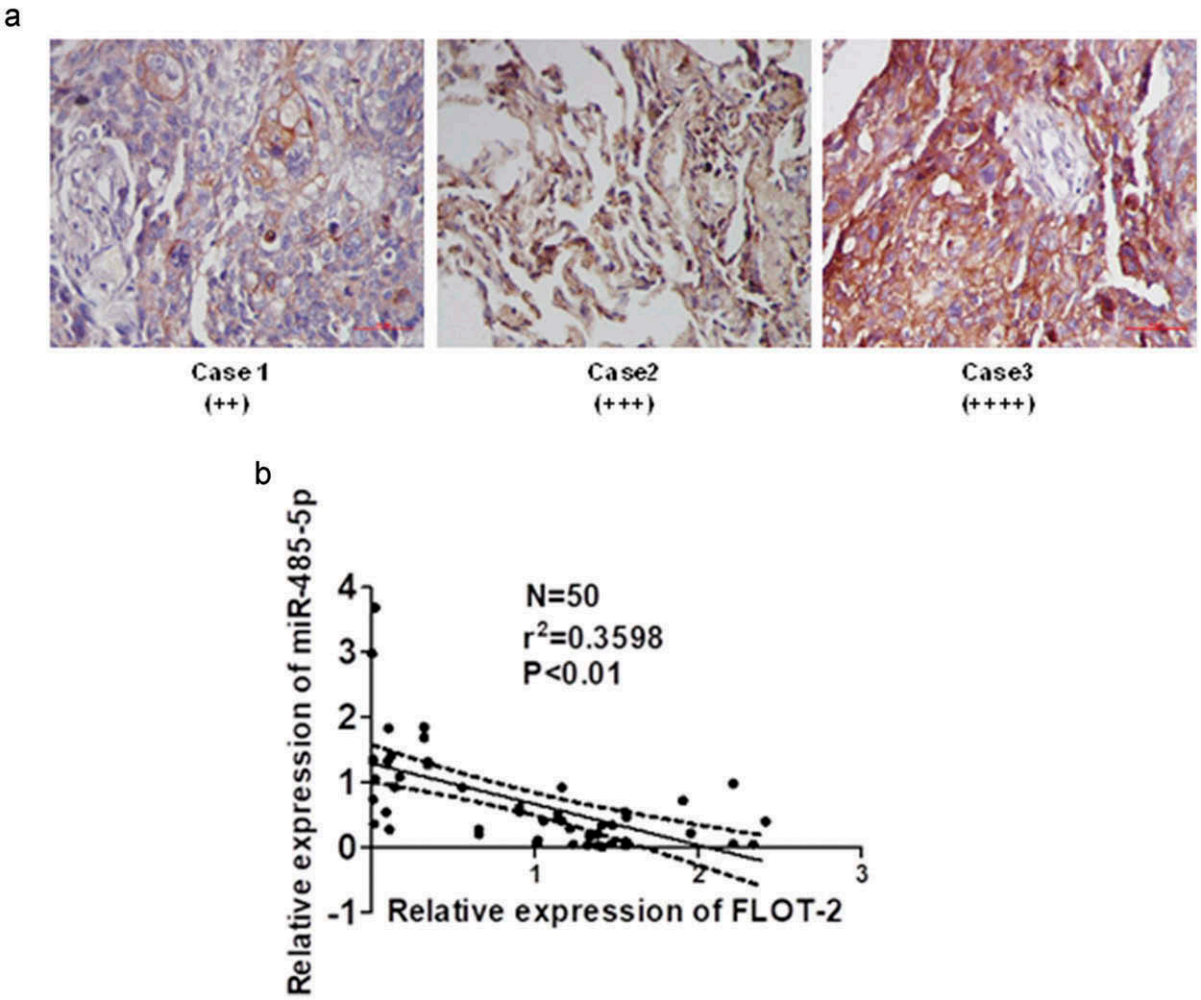
Relationship between MiR-485-5p level and FLOT2 expression in SCLC tissues. (a), immunohistochemical staining of FLOT2 on the tissue sections of SCLC; (b), negative correlation between miR-485-5p and FLOT2 mRNA level; FLOT2 mRNA and miR-485-5p were measured by RT-qPCR, and Pearson correlation analysis shows a negative correlation between miR-485-5p and FLOT2 mRNA level (n = 50, r = −0.5991, p < 0.01).

To elucidate the relationship between miR-485-5p and FLOT2 in cancer tissues of SCLC patients, FLOT2 protein was detected using immunostaining, and miR-485-5p and FLOT2 mRNA were quantified using qPCR. Immunostaining showed that FLOT2 expression was markedly increased in cancer tissues of SCLC patients (Figure 8(a)). miR-485-5p expression level was significantly negatively correlated with FLOT2 mRNA levels (n = 50, r = −0.5991, p < 0.01) in SCLC (Figure 8(b)). These results suggested that miR485-5p targeted FLOT2 in cancer tissues of SCLC patients.

## Discussion

In this study, it was found that miR-485-5p expression was decreased in SCLC tissues compared with the normal tissues. In addition, synthetic miR-485-5p mimics inhibited the growth, migration and invasion of SCLC cells *in vitro*. Furthermore, FLOT2 was upregulated in SCLC, and was a direct target of miR-485-5p in SCLC cells. These findings demonstrate that miR-485-5p acts as a tumor suppressor by directly targeting FLOT2.

The expression level of miR-485-5p was lower in human SCLC tissues than normal tissues, which is in agreement with the observations in human NSCLC by Huang *et al*. [31]. The elevated level of miR-485-5p in the NCI-H446 and NCI-H1688 cells transfected with miR-485-5p mimics and the reduced level in the NCI-H446 and NCI-H1688 cells transfected with miR-485-5p inhibitor indicate that models of NCI-H446 and NCI-H1688 cells with miR-485-5p overexpression and knockdown were successfully achieved. The miR-485-5p overexpression mediated reduction in the proliferation, migration and invasion and miR-485-5p silencing mediated increase in the proliferation, migration and invasion in NCI-H446 and NCI-H1688 cells, and support the observation that miR-485-5p targets specific genes to regulate proliferation, migration and metastasis in SCLC [33,34]. These observations demonstrated that miR-485-5p acts as a tumor suppressor, inhibiting growth and metastasis, in SCLC.

FLOT2 is a major protein on lipid rafts, important for non-caveolar raft formation and associated with the development and progression of cancer [34,35]. The expression of miR-485-5p was negatively correlated with FLOT2 mRNA expression in SCLC tissues. Bioinformatics analysis using miRNA target prediction algorithms miRanda (www.microrna.org) and RNAhybrid (http://bibiserv.techfak.uni-bielefeld.de/rnahybrid/submission.html) revealed a miR-485-5p-binding site in the 3′-UTR of FLOT2, and predicted it was a potential target gene of miR-485-5p. The decreased protein expression of FLOT2 in miR-485-5p mimic-transfected NCI-H446 and NCI-H1688 cells and increased protein expression of FLOT2 in miR-485-5p inhibitor-transfected NCI-H446 and NCI-H1688 cells indicated that miR-485-5p reduced the production of FLOT2 protein. Co-transfection of NCI-H446 cells with the wild-type psiCHECK2-FLOT2-3′-UTR and miR-485-5p mimics decreased the luciferase activity of the reporter gene, whereas co-transfection of the cells with mutant psiCHECK2-FLOT2-3′-UTR-mut and miR-485-5p mimics abolished the inhibitory effect of miR-485-5p on luciferase activity. These results indicate that miR-485-5p reduces the translation product of FLOT2 mRNA via acting on its 3ʹ-UTR, and support the observations that microRNAs can regulate the expression level of FLOT2 [36], the genes in FLOT2 related growth factor receptors linked signal transduction pathways [37,38], and influence the development and progression of cancer. It was also showed that miR-485-5p influenced FLOT1 in NCI-H1688 cells but not in NCI-H446 cells. The mechanisms underlying the difference in the effect of miR-485-5p on FLOT1 between the two cell lines was not clear.

In conclusion, miR-485-5p was downregulated in SCLC tissues, which was negatively correlated with the mRNA expression of FLOT2. miR-485-5p inhibited SCLC cell proliferation, migration and invasion by targeting FLOT2. The restoration of miR-485-5p expression may be an attractive strategy for SCLC therapy.
